# Polyglutamylated Tubulin Binding Protein C1orf96/CSAP Is Involved in Microtubule Stabilization in Mitotic Spindles

**DOI:** 10.1371/journal.pone.0142798

**Published:** 2015-11-12

**Authors:** Shinya Ohta, Mayako Hamada, Nobuko Sato, Iyo Toramoto

**Affiliations:** From the Center for Innovative and Translational Medicine, Medical School, Kochi University, Kohasu, Oko-cho, Nankoku, Kochi, Japan; Virginia Tech, UNITED STATES

## Abstract

The centrosome-associated C1orf96/Centriole, Cilia and Spindle-Associated Protein (CSAP) targets polyglutamylated tubulin in mitotic microtubules (MTs). Loss of CSAP causes critical defects in brain development; however, it is unclear how CSAP association with MTs affects mitosis progression. In this study, we explored the molecular mechanisms of the interaction of CSAP with mitotic spindles. Loss of CSAP caused MT instability in mitotic spindles and resulted in mislocalization of Nuclear protein that associates with the Mitotic Apparatus (NuMA), with defective MT dynamics. Thus, CSAP overload in the spindles caused extensive MT stabilization and recruitment of NuMA. Moreover, MT stabilization by CSAP led to high levels of polyglutamylation on MTs. MT depolymerization by cold or nocodazole treatment was inhibited by CSAP binding. Live-cell imaging analysis suggested that CSAP-dependent MT-stabilization led to centrosome-free MT aster formation immediately upon nuclear envelope breakdown without γ-tubulin. We therefore propose that CSAP associates with MTs around centrosomes to stabilize MTs during mitosis, ensuring proper bipolar spindle formation and maintenance.

## Introduction

C1orf96, termed as Centriole, Cilia and Spindle-Associated Protein (CSAP or CCSAP), is required for proper cilia beating and is targeted to polyglutamylated microtubules (MTs; [1]). Immunoelectron microscopy of CCSAP in centrosomes has shown primary localization to the MT cylinder walls and secondary localization in the centriole lumen. CSAP is required for proper zebrafish development and lateral asymmetry in the brain [1–3]. Polyglutamylation at the C-terminus of α- and γ-tubulin accumulates in neuronal cultures and in the brain during development [4–7]. Tubulin tyrosine ligase-like (TTLL) proteins show polyglutamylase activity for tubulin in mammalian cells [8–10]. Polyglutamylation of MT regulates its association with microtubule-associated proteins (MAPs) and motor proteins because the C-terminal domains of α- and β-tubulin are exposed on the outer MT surfaces [9,11–13]. Extensive polyglutamylation of tubulin leads to MT stability [14–16]. However, the molecular function of microtubule polyglutamylation and CSAP binding to polyglutamylated MTs during mitosis remains unclear.

Accurate spindle formation is essential for proper segregation of chromosomes during cell division. The bipolar mitotic spindle is composed of MTs organized in polar structures, with minus ends anchored to centrosomes and plus ends emanating out into the space between the two spindle poles. These MTs are dynamic and continuously shift between a state of polymerization (growth) and de-polymerization (shrinkage), aiding in the capture of kinetochores on mitotic chromosomes [17,18]. A subclass of MT plus-ends from opposite poles can also participate in antiparallel interactions in the midzone to stabilize spindle assemblies [19–23].

The γ-tubulin ring complex (γ-TuRC) occupies the MT minus ends and acts as a platform for mitotic spindle formation [24]. The pericentriolar protein CDK5RAP2 is required for maintenance of centriole engagement and cohesion [25,26]. Moreover, CDK5RAP2 is recruited to the centrosomes by dynein and mediates spindle pole attachment during mitosis [27,28]. Nuclear protein that associates with the Mitotic Apparatus (NuMA) interacts with cytoplasmic dynein and dynactin, and is transported toward MT minus ends [29–33]. Therefore, motor proteins accumulate in the pericentrosomal region and generate pulling forces on the astral MTs [34,35]. Astral MTs grow all around the centrosomes and emanate away from the mitotic spindle and towards the cell cortex, thereby regulating spindle position in the mitotic cell. Correct centrosome positioning in the mitotic cell and MT nucleation at these sites are extremely important for maintaining normal spindle shape and structure [19]. We recently demonstrated that the mitotic spindle protein CENP-32 is required for centrosome association with the spindle poles and maintains centrosomal dominance in bipolar spindle assembly [36]. Moreover, it has been reported that taxol treatment induces the formation of nocodazole-resistant MTs and leads to formation of centrosome-free MT asters or poles [37], which can also be induced by other MT stabilization drugs [38,39]. Centrosome-free MT asters have been studied and characterized by many groups [40,41].

Here, we report that CSAP blocks MT depolymerization by cold and nocodazole treatments. In addition, CSAP overexpression or depletion promotes multipolar spindle formation by promoting mislocalization of pericentrosomal proteins. We conclude that CSAP co-localizes with polyglutamylated MTs to promote MT stabilization and regulate bipolar spindle formation in mitosis.

## Materials and Methods

### Cell culture

U2OS and HeLa cells (Kyoto) in the exponential growth phase were seeded onto coverslips and grown overnight in DMEM (Wako: 044–29765) with 10% FBS (Biowest: S1610-500), 100 U/mL penicillin, and 100 μg/mL streptomycin (Wako: 168–23191) at 37°C under a 5% CO_2_ atmosphere in a humid incubator.

### Transfection plasmids and siRNA oligos

To overexpress GFP-CSAP, TrAP-CSAP, or TrAP (control), expression vectors were transfected into U2OS or HeLa cells at ~60% confluence using Lipofectamine LTX Reagent with PLUS Reagent (Life Technologies: A12621). Cells were maintained for 24 h before fixation. siRNA was added to U2OS cells at 60% confluence by transfection with Lipofectamine RNAiMAX Transfection Reagent (Life Technologies: 13778100) in complete medium without antibiotics (Control: AACGUACGCGGAAUACUUCGAdTdT; CCSAP: Thermo Fisher Scientific siGENOME SMARTpool: M-016008-00). Cells were cultured for 72 h before fixation.

### Indirect immunofluorescence microscopy

Cells were fixed for 7 min with 4% (v/v) paraformaldehyde (Wako: 163–20145) in PBS or for 2 min in cold methanol. After permeabilization with 0.15% (v/v) Triton X-100 in PBS, coverslips were blocked with 1% (v/v) BSA in PBS. Cells were probed with antibodies against the following: pericentrin (1:1000, ab4448, Abcam), γ-tubulin (1:1000, AK15, Sigma; 1:1000, GTU-88, Sigma), α-tubulin (1:2000, B512, Sigma; 1:200, ab89984, Abcam), CDK5RAP2 (1:1000, #06–1398, Millipore), Aurora A (1:100, #4718, CST), NuMA (1:100, #3888, CST), SBP (1:200 [42]), and polyglutamylation modification (1:1000, GT335, AdipoGen). Cells were washed three times with PBS for 5 min, Alexa-conjugated secondary antibodies were applied at 1:600, and the DNA was counterstained with DAPI at 0.1 μg/mL. Single-confocal-plane images were obtained using an Olympus FV1000 based on an IX81 confocal microscope system with a UPlan SApo 60×/1.35 oil immersion objective lens (Olympus) and FV10-SAW2.1 software (Olympus). The images were Kalman filtered to suppress noise. Single-focal-plane images for fluorescence quantification were obtained using an Olympus BX53 microscope with a UPlan SApo 60×/1.35 oil immersion objective lens and a DP73 CCD camera (Olympus), with CellSens standard 1.7 software (Olympus). For quantitation of α-tubulin, γ-tubulin, Aurora A, CDK5RAP2, NuMA, and polyglutamylation signals, mean fluorescence intensities on the spindle were measured using ImageJ 1.46r software. Background fluorescence intensity, which was determined by multiplying the mean intensity in the region next to the target cell with an equivalent area of the target fluorescence signal, was subtracted from the target fluorescence intensity. For normalization of NuMA or polyglutamylation signal intensities, the region of interest (ROI) covering pixels of the α-tubulin fluorescence signal from the spindle poles was used as the mitotic aster region. Fluorescence signal intensities of NuMA or polyglutamylation in this ROI were normalized by division with α-tubulin fluorescence signal intensities in the same ROI before averaging. For statistical analysis of the data, we used a paired Student’s t-test.

### MT regrowth assay

For MT regrowth assay, cells were placed in ice-cold DMEM medium for 30 min, after which time the ice-cold medium was replaced with DMEM medium pre-warmed at 37°C. At the indicated time points (0, 3, or 25 min) after medium replacement, cells were fixed for 2 min in cold methanol and immunostained as described above.

### Time-lapse fluorescence microscopy

The H2B-mRFP fusion protein expression plasmid, pRFP-C-H2B, and the GFP-α-tubulin fusion protein expression plasmid, pEGFP-N-tubulin, were transfected into U2OS cells using Lipofectamine LTX. Stable clonal U2OS cell lines expressing these fusion proteins were generated and maintained with 400 μg/mL G418 (Wako: 074–05963). For capturing images, cells were grown at 37°C in DMEM without phenol red (Wako: 044–32955) and with 10% FBS under a 5% CO_2_ atmosphere [36]. Image capture began 12 h after transfection with the CSAP overexpression plasmid as indicated above and continued for up to 36 h. Time-lapse images were acquired every 5 min in a 5% CO_2_/37 °C chamber using a BioStation IM system (Nikon).

### Immunoblotting

The primary antibodies were rabbit anti-CSAP at 1:500 (ab102623; Abcam) and anti-α-tubulin at 1:1000 (B-5-1-2; Sigma-Aldrich). The secondary antibodies were IRDye 800CW donkey anti-rabbit IgG at 1:10000 (926–32211; Li-Cor Biosciences) or anti-mouse IgG at 1:10000 (926–32210; Li-Cor Biosciences). The immune complexes were detected by using an Odyssey CLx Infrared Imaging System (Li-Cor Biosciences) and ImageStudio 5.2 software (Li-Cor Biosciences).

## Results

### Overexpression of C1orf96 promotes multipolar spindle formation in mitosis

**Fig 1:**
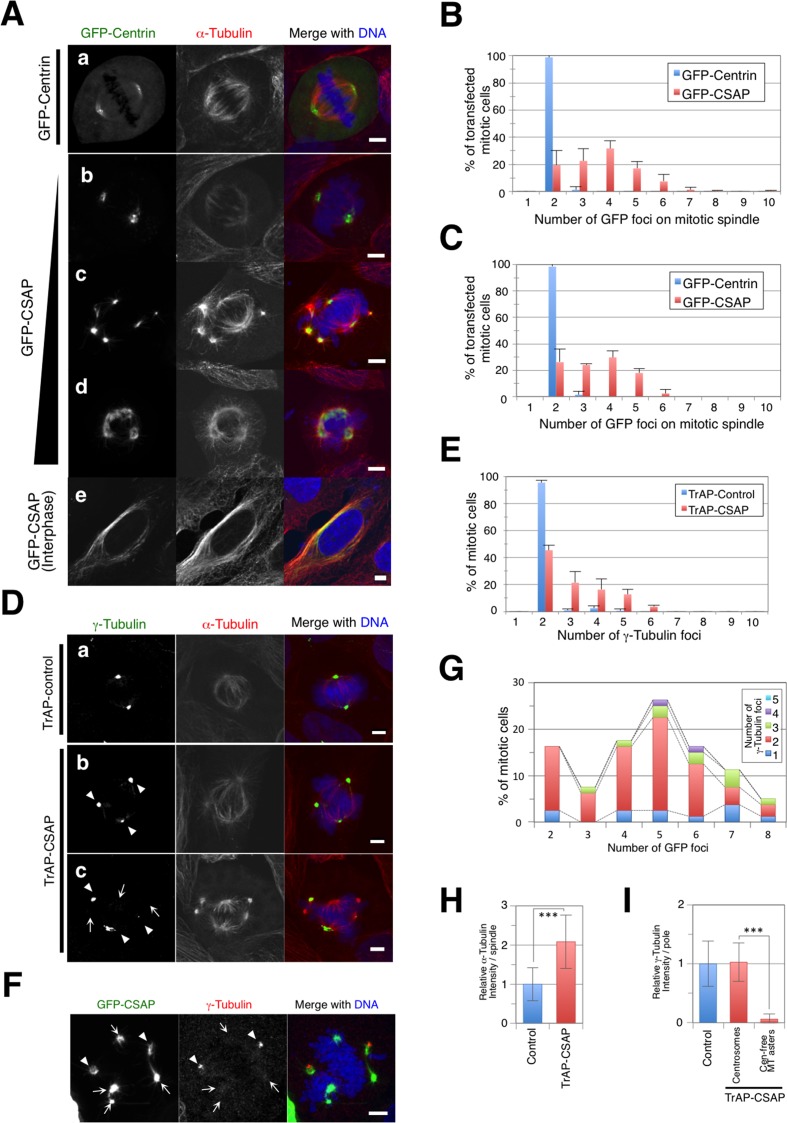
Overexpression of CSAP promotes the formation of centrosome-free MT asters on mitotic spindles. **(A)** U2OS cells transiently expressing GFP-CSAP (a) or GFP-centrin (mitosis, b-d; interphase, e). Cells were stained for α-tubulin (Red) and DNA (Blue). GFP-CSAP localizes to centrosomes; cells overexpressing GFP-CSAP show multiple GFP foci on the mitotic spindles. Scale bar, 5 μm. Over-expression is indicated by the graph on the left side. **(B, C)** Proportion of U2OS **(B)** or HeLa cells **(C)** transiently expressing GFP-centrin or GFP-CSAP with the number of GFP foci on mitotic spindles. Data represent the mean ± SD of three experiments. **(D)** U2OS cells transiently expressing TrAP (a) or TrAP-CSAP (b, c). Cells were stained for α-tubulin (red), γ-tubulin (green), and DNA (blue). Microtubule asters with γ-tubulin (arrowheads) and without γ-tubulin (arrows) are indicated. **(E)** Proportion of U2OS cells transiently expressing TrAP or TrAP-CSAP with the number of γ-tubulin foci on mitotic spindles. Data represent the mean ± SD of three experiments. **(F)** U2OS cells transiently expressing GFP-CSAP. The cells were stained forα-tubulin (red), γ-tubulin (green), and DNA (blue). Microtubule asters with γ-tubulin (arrowheads) and without γ-tubulin (arrows) are indicated. **(G)** Proportion of U2OS cells transiently expressing GFP-CSAP with the number of GFP-CSAP foci vs. γ-tubulin foci on mitotic spindles. **(H, I)** Comparison of α-tubulin **(H)** and γ-tubulin **(I)** on mitotic spindles in cells transiently expressing control (blue) or TrAP-CSAP (red). In **(I)**, two types of γ-tubulin foci at centrosomes and centrosome-free MT asters are separately shown. Data represent the mean ± SD relative intensity. *** indicates p < 0.005 (n = ~40).

GFP-CSAP in transiently transfected U2OS cells was primarily localized around the centrosomes during mitosis, which is consistent with previous reports (Fig 1A-b, [1]). Additionally, in interphase, it was strongly associated with microtubules (Fig 1A-e). We also found more than three GFP foci on mitotic spindles in GFP-CSAP overexpressing cells (81%; Fig 1B); this phenotype was dependent on the extent of GFP-CSAP overexpression (Fig 1A-b, 1A-c and 1A-d). We observed MT elongation from each GFP focus (Fig 1A-c and 1A-d). These results significantly differed from those derived from transient transfection with GFP-centrin, a known centrosomal protein (1.3%; Fig 1A and 1B). The effect was reproducible in transiently transfected HeLa (Kyoto) cells (GFP-CSAP, 74%; GFP-centrin, 1.4%; Fig 1C). Thus, overexpression of C1orf96/CSAP induced multipolar spindle formation in mitosis.

### Formation of spindle poles without centrosomal components is promoted by CSAP overexpression

Next, we investigated the localization of triple affinity peptide (TrAP: his6, Streptavidin binding peptide, and S tag)-tagged CSAP in mitotic U2OS cells. The TrAP tag is much smaller than the GFP tag and produces less steric hindrance, enabling CSAP to approach the endogenous protein. Indirect immunofluorescence of α-tubulin and γ-tubulin was used to characterize the number of mitotic centrosomes; 44% of mitotic cells contained more than three spindle poles stained by γ-tubulin after transfection with the TrAP-CSAP expression vector (Fig 1D-b, 1D-c and 1E), which was much higher than the 4% observed in control cells (Fig 1D-a and 1E). We suggest that CSAP overexpression causes multipolar spindle formation in mitosis and inhibits proper bipolar spindle formation; thus, proper expression of CSAP is required for normal mitotic progression.

The percentage of multipolar spindles in mitotic cells overexpressing GFP-CSAP was nearly twice that in cells overexpressing TrAP-CSAP (GFP-CSAP, 81% in Fig 1F; TrAP-CSAP, 44% in Fig 1E). Immunofluorescence analysis of TrAP-CSAP-overexpressing cells showed MT asters and foci without γ-tubulin co-localization (Fig 1D). Moreover, γ-tubulin quantification analysis significantly showed two types of poles. One contained γ-tubulin at an unchanged level, whereas in the other type, the amount of γ-tubulin was below the measurable limit (Fig 1I). We then looked for overlap of GFP-CSAP foci on mitotic spindles with mitotic centrosomes containing γ-tubulin. Only 35% of GFP foci on the spindles overlapped with centrosomes in mitotic cells overexpressing GFP-CSAP (Fig 1G). Although the mitotic cells showed multipolar spindles, 71% of them contained two γ-tubulin foci (Fig 1G). Centrosome-free spindle pole formation caused by MT stabilization has been reported in previous studies [37,40]. Our data suggest that excess CSAP on mitotic spindles promotes formation of centrosome-free MT asters/poles. Additionally, α-tubulin staining was stronger in mitotic cells containing centrosome-free MT asters (Fig 1H).

### Time-lapse analysis of centrosome-free MT aster formation by over-expression of CSAP

**Fig 2:**
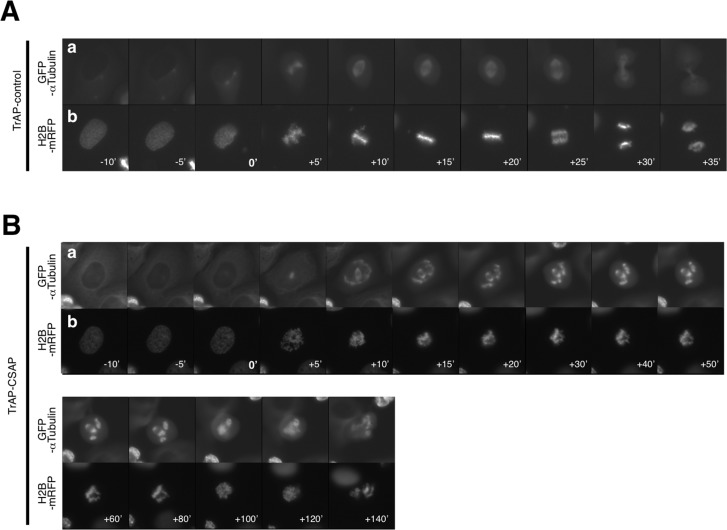
Time-lapse analysis of CSAP-overexpressing cells during mitosis. Panels summarize time-lapse recordings of control U2OS **(A, S1 Movie)** and TrAP-CSAP-overexpressing cells **(B, S2 Movie)** expressing GFP–α-tubulin (upper) and H2B-mRFP (lower). Times are minutes after NEBD (0’).

Time-lapse microscopy was also used to analyze centrosome-free MT aster formation in U2OS cells overexpressing TrAP-CSAP and GFP–α-tubulin fusion proteins. In the absence of TrAP-CSAP overexpression, bipolar spindle formation began immediately after nuclear envelope breakdown (NEBD), and the onset of anaphase occurred after 22.9 ± 4.9 min (n = 12; Fig 2A, S1 Movie). In cells overexpressing TrAP-CSAP, no centrosome-free MT asters were observed prior to NEBD; after NEBD, centrosome-free MT asters were observed as soon as spindle formation occurred (Fig 2B, S2 Movie; 10). The timing of centrosome-free MT aster formation was similar in CSAP-overexpressing cells (S2–S4 Movies). This result suggests that the centrosome-free MT aster-forming activity of CSAP occurs only during mitosis. After centrosome-free MT formation, we were unable to observe α-tubulin dynamics over time (Fig 2B, S2 Movie; from 30’ to 80’) and speculated that CSAP binding to MTs inhibits depolymerization. We next asked whether multipolar and centrosome-free MT aster formation in CSAP-overexpressing cells may have been caused by fragmentation of pericentrosomal material (PCM, [43]) or by de novo multi-polar/aster formation. Under live-cell observation, we observed MT nucleation from the nuclear envelope and multipolar formation immediately after NEBD, but did not observe any multipolarity resulting from PCM fragmentation (S2–S4 Movies). Thus, we concluded that CSAP overexpression-induced multipolarity is not caused by PCM fragmentation, but rather that there is a possibility that it occurs de novo via a γ-tubulin-independent mechanism.

### CSAP recruits pericentrosomal components on mitotic spindles

**Fig 3:**
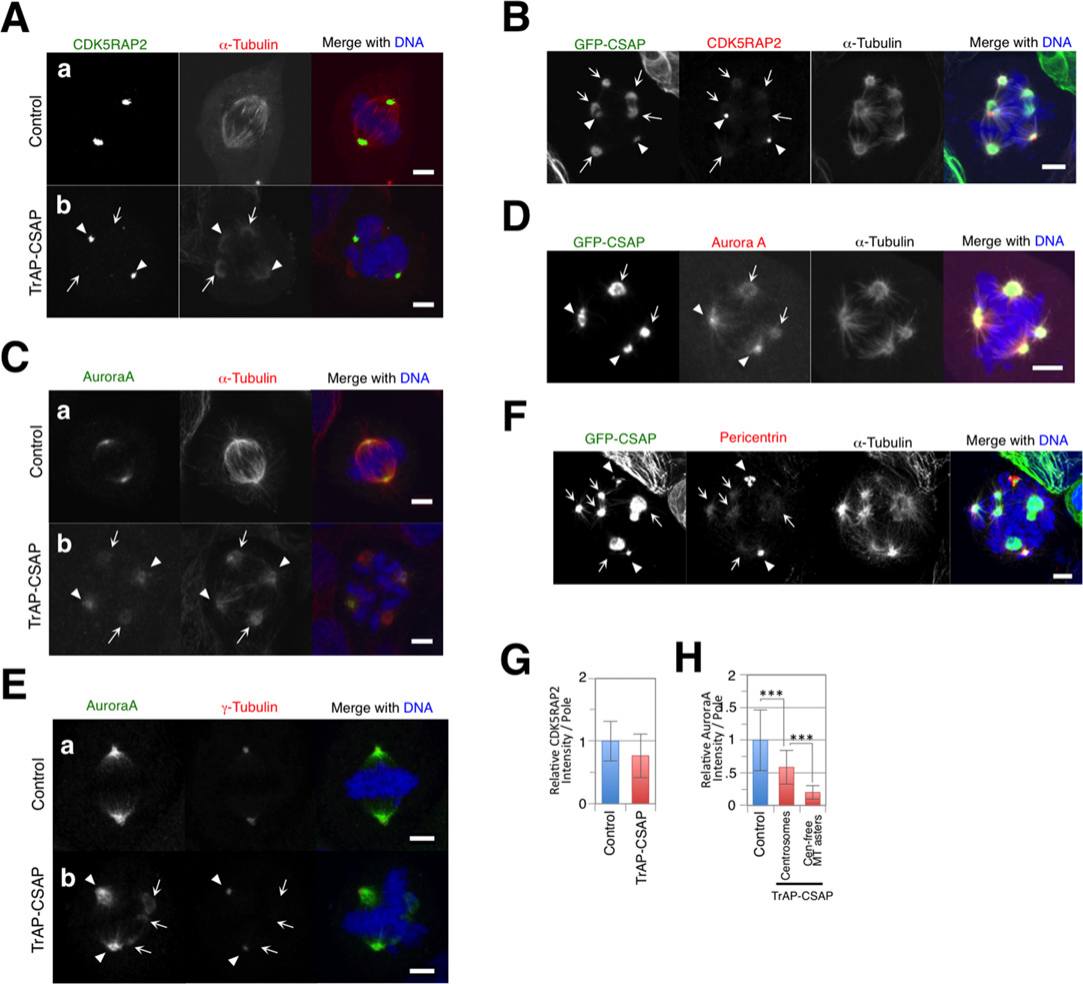
Pericentrosomal material composition of centrosome-free MT asters. **(A, C)** Mitotic U2OS cells transiently expressing control (a) and TrAP-CSAP (b). Cells were stained for α-tubulin (Red), DNA (Blue), and CDK5RAP2 (**A**; Green) or Aurora A (**C**; Green). **(B, D, E)** Mitotic U2OS cells transiently expressing GFP-CSAP (Green). Cells were stained for α-tubulin (White), DNA (Blue), and CDK5RAP2 (**B**; Red), Aurora A (**D**; Red), or pericentrin (**E**; Red). **(F)** Mitotic U2OS cells transiently expressing control (a) and TrAP-CSAP (b). The cells were stained for γ-tubulin (Red), DNA (Blue), and Aurora A. Scale bar, 5 μm. **(G, H)** Comparison of CDK5RAP2 **(G)** and Aurora A **(H)** at mitotic spindle poles in cells transiently expressing control (blue) or TrAP-CSAP (red). In **(H)**, two types of Aurora A foci at centrosomes and centrosome-free MT asters are separately shown. Data represent the mean ± SD relative intensity. *** indicates p < 0.005 (n = ~30).

**Fig 4:**
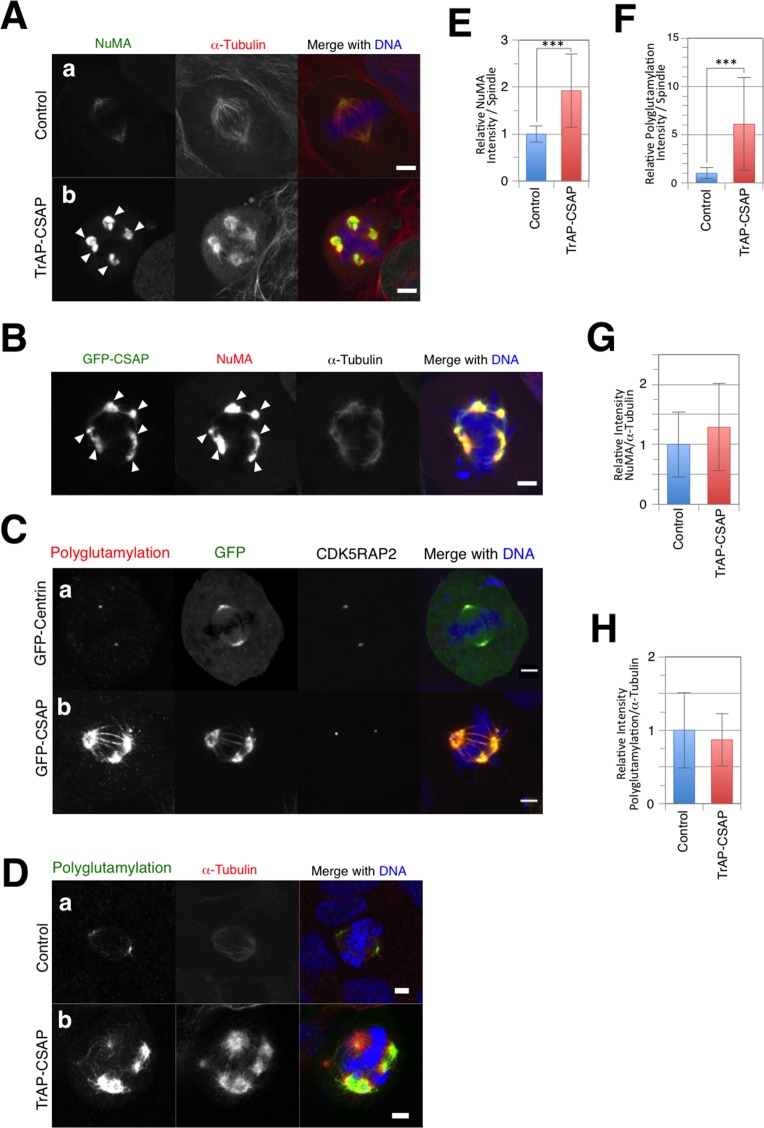
Increasing NuMA and polyglutamylation on mitotic spindles containing centrosome-free MT asters. **(A, D)** Mitotic U2OS cells transiently expressing control (a) and TrAP-CSAP (b). Cells were stained for α-tubulin (Red), DNA (Blue), and NuMA (Green). **(B)** Mitotic U2OS cells transiently expressing GFP-CSAP (**A**; Green) or polyglutamylation (**D**). Cells were stained for NuMA (Red), α-tubulin (White), and DNA (Blue). **(C)** Mitotic U2OS cells transiently expressing GFP-CSAP (Green). Cells were stained for polyglutamylation (Red), CDK5RAP2 (White), and DNA (Blue). Scale bar, 5 μm. **(E, F)** Comparison of NuMA **(E)** and polyglutamylation **(F)** on mitotic spindles in cells transiently expressing control (blue) or TrAP-CSAP (red). **(G, H)** Comparison of α-tubulin vs. NuMA **(G)** or polyglutamylation **(H)** on the spindles around the centrosomes in transiently expressing control (blue) or on centrosome-free MT asters in cells transiently expressing TrAP-CSAP (red). Data represent the mean ± SD relative intensity. *** indicates p < 0.005 (n = ~30).

We demonstrated γ-tubulin-independent centrosome-free MT aster formation in CSAP-overexpressing mitotic cells, suggesting that the protein composition of centrosome-free MT asters differs from that of proper spindle poles and centrosomes. Immunofluorescence labeling of various centrosomal proteins in TrAP-CSAP-overexpressing cells was used to assess the composition of spindle poles and centrosome-free MT asters. CDK5RAP2 maintains the pericentriolar material (PCM) and spindle pole bodies around centrioles during mitosis in *Drosophila melanogaster*, fission yeast, and vertebrates [26,28,44,45]. Two significant CDK5RAP foci were frequently observed in TrAP-CSAP overexpressing mitotic cells, even those with more than two spindle poles (Fig 3B; arrowheads), and not all GFP foci maintained CDK5RAP2 in GFP-CSAP over-expressing mitotic cells (Fig 3B; arrows). Moreover, CDK5RAP2 accumulation was unaltered by TrAP-CSAP overexpression (Fig 3G). Aurora A is required for centrosome maturation and bipolar spindle formation [46]. Cells expressing TrAP-CSAP exhibited two patterns of Aurora A staining on mitotic spindles (Fig 3C, arrows and arrowheads), both of which co-localized with CSAP foci (Fig 3D). γ-Tubulin was present at stronger-stained Aurora A foci (Fig 3E, arrowheads), but not at weaker-stained foci (Fig 3E, arrows). Thus, Aurora A recruitment to centrosomes was normal in CSAP-overexpressing cells, and additional recruitment to centrosome-free MT asters also occurred. Pericentrin/kendrin generates the pericentriolar material (PCM) via interaction with γ-tubulin and the motor protein dynein [47,48]. Pericentrin exhibited the same staining pattern as Aurora A (Fig 3F). In contrast, NuMA accumulated at centrosomes and centrosome-free MT asters in TrAP-CSAP-overexpressing cells (Fig 4A arrowheads) and co-localized with GFP-CSAP at centrosome-free MT asters (Fig 4B, arrowheads); the amount of aster-associated NuMA was significantly larger in TrAP-CSAP-overexpressing cells compared to controls (Fig 4E). However, NuMA fluorescence signals normalized by α-tubulin signals in individual mitotic cells did not differ in CSAP-overexpressing cells compared to controls (Fig 4G). Therefore, we propose that CSAP increases the amount of NuMA and other centrosomal proteins associated with mitotic spindle poles through its microtubule stabilization function; this may result in the formation and maintenance of centrosome-free MT asters during mitosis in CSAP-overexpressing cells.

### MT stabilization by CSAP enhances its polyglutamylation on mitotic spindles

CSAP is recruited to polyglutamylated α- and β-tubulin on mitotic spindles [1]. Here, we investigated polyglutamylated MT levels in the spindles of mitotic cells containing centrosome-free MT asters by immunofluorescence microscopy with a monoclonal anti-polyglutamylation antibody (GT335). In the control cells, polyglutamylation was observed around spindle poles (Fig 4C-a and 4D-a); however, most of the spindles were more strongly polyglutamylated in mitotic cells because of overexpression of CSAP (Fig 4C-b and 4F). Moreover, cells overexpressing GFP-CSAP presented centrosome-free MT aster polyglutamylation where CSAP was bound to MTs on the mitotic spindles (Fig 4C-b). These results suggest that binding of CSAP to MTs increases the polyglutamylation on the mitotic spindles. We quantitatively compared polyglutamylation fluorescence signals normalized by α-tubulin signals around spindle poles in control cells and on centrosome-free MT asters in TrAP-CSAP overexpressing cells. Polyglutamylation accumulation on MTs in the mitotic asters was unaltered by TrAP-CSAP overexpression (Fig 4H), which suggests that the increase in polyglutamylation was a result of increased MT stability.

### CSAP is critical for the centrosomal localization of NuMA

**Fig 5:**
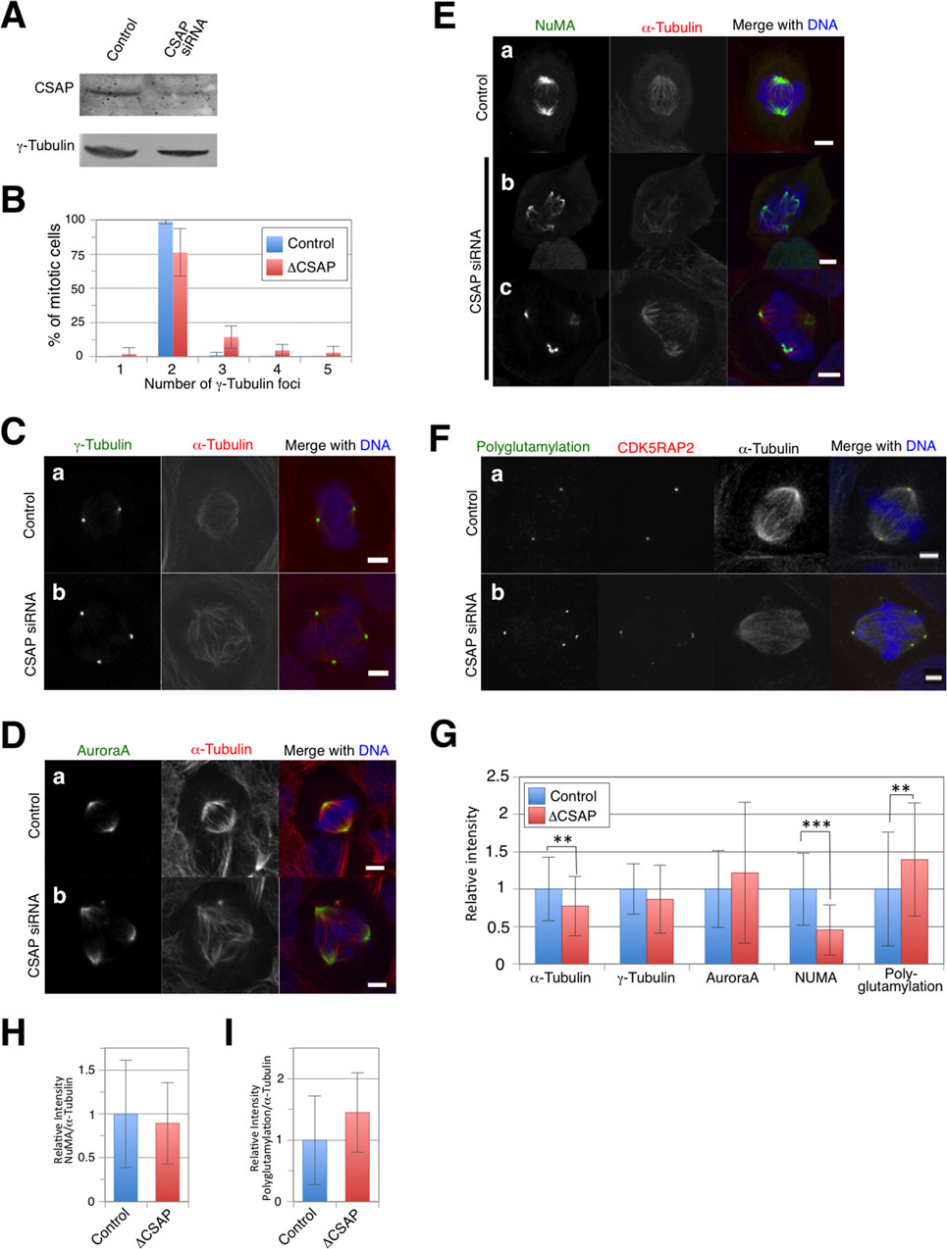
CSAP depletion causes NuMA mislocalization during mitosis. **(A)** At 72 h after transfection with control or pooled siRNAs for CSAP, U2OS cells were lysed and immunoblotted with anti-CSAP antibody. **(B)** Proportion of control or CSAP siRNA-transfected U2OS cells with γ-tubulin foci on mitotic spindles. Data represent the mean ± SD of three experiments. **(C, D, E)** Control (a) or CSAP (b, c) siRNA-transfected U2OS cells were fixed and immunostained for α-tubulin (Red), DNA (Blue), and γ-tubulin (**C**; Green), Aurora A (**D**; Green), or NuMA (**E**; Green). **(F)** Control (a) and CSAP (b) siRNA-transfected U2OS cells were fixed and immunostained for polyglutamylation (Green), CDK5RAP2 (Red), α-tubulin (white), and DNA (Blue). Scale bar, 5 μm. **(G)** Comparison of α-tubulin, γ-tubulin, Aurora A, NuMA, and polyglutamylation staining intensity on mitotic spindles in control (blue) or CSAP-depleted (red) cells. **(H, I)** Comparison of α-tubulin vs. NuMA **(H)** or polyglutamylation **(I)** immunostaining on the spindles around the centrosomes in control (blue) or CSAP-depleted (red) cells. Data represent the mean ± SD relative intensity. ** and *** indicate p < 0.01 and p < 0.005, respectively. (n = ~30).

To test our hypothesis that CSAP recruits NuMA and Aurora A around centrosomes, we investigated the phenotype of CSAP depletion by siRNA. CSAP levels at 72 h post-transfection were significantly decreased (Fig 5A). Increased multipolar spindle formation in CSAP-depleted cells was observed by counting centrosomes via indirect immunofluorescence targeting α-tubulin and γ-tubulin (ΔCCSAP 21%, control 1%; Fig 4B). These results suggest that CSAP is involved in proper bi-polar spindle formation in mitosis. Because the γ-tubulin signal was detected in spindle poles in CSAP-depleted multipolar cells, this multipolarity is mechanistically different from that induced by CSAP overexpression. Quantitative analysis showed no significant change in γ-tubulin levels on centrosomes after multipolar spindle formation in CSAP-depleted mitotic cells (Fig 5C and 5G). Aurora A levels also remained unchanged after depletion (Fig 5D and 5G), although NuMA accumulation on mitotic spindles decreased (Fig 5E and 5G). The NuMA signal largely disappeared from the spindle distant from the centrosomes, and faint NuMA staining was frequently observed (Fig 5E-b). Thus, CSAP is required for proper NuMA localization on mitotic spindles near centrosomes. In contrast, no significant change was observed in the polyglutamylation at the centrosomes after CSAP depletion (Fig 5F and 5G), suggesting that CSAP is not essential for this phenomenon. The α-tubulin signal substantially decreased after CSAP depletion (Fig 5C–5G). Moreover, NuMA fluorescence signals normalized by α-tubulin signals in individual mitotic cells did not change after CSAP depletion (Fig 5H). Finally, the polyglutamylation fluorescence signals normalized by α-tubulin signals slightly increased after CSAP depletion (Fig 5I).

### MT regrowth assay in CSAP-overexpressing cells

**Fig 6:**
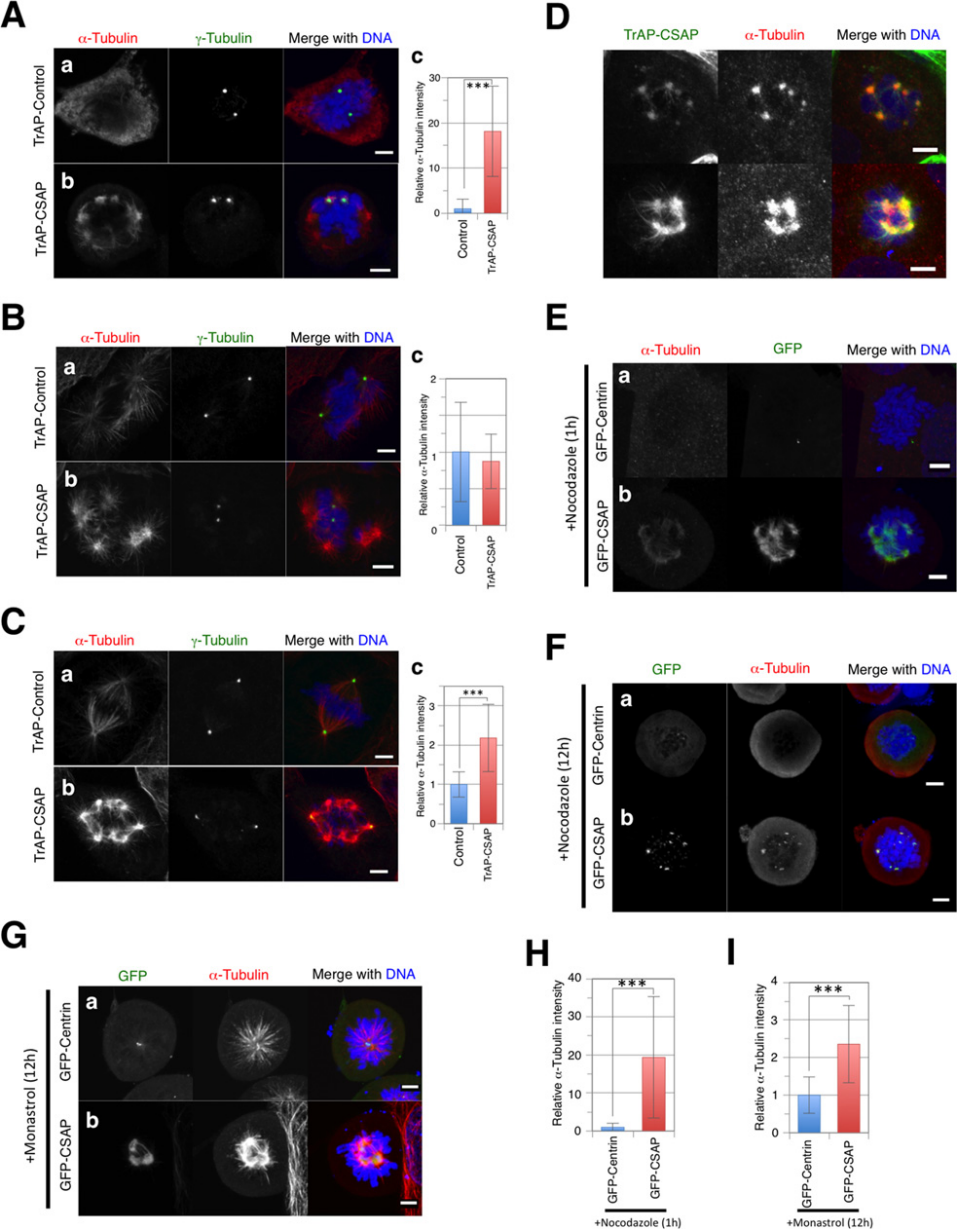
MT regrowth assay in CSAP over-expressing cells. **(A, B, C)** MT regrowth assay of mitotic U2OS cells transiently over-expressing TrAP-centrin (a) or GFP-CSAP (b). After recovery from cold treatment for 0 min **(A)**, 3 min **(B)**, and 25 min **(C)**, the cells were stained for α-tubulin (Red), γ-tubulin (Green), and DNA (Blue). (c) Quantitative comparisons of α-tubulin are shown. Data represent the mean ± SD relative intensity. ** and *** indicate p < 0.01 and p < 0.005, respectively. **(D)** Mitotic U2OS cells transiently over-expressing TrAP-CSAP after cold treatment. Cells were stained for TrAP-CSAP (Green), γ-tubulin (Red), and DNA (Blue). **(E)** Images of mitotic U2OS cells transiently over-expressing GFP-Centrin (a) or GFP-CSAP (b) after 1 h of treatment with nocodazole before fixation. The cells were stained for α-tubulin (Red) and DNA (Blue). **(F, G)** Mitotic U2OS cells transiently over-expressing GFP-Centrin (a; Green) or GFP-CSAP (b) with nocodazole **(F)** or monastrol **(G)** for 12 h. Cells were stained for α-tubulin (Red) and DNA (Blue). Scale bar, 5 μm. **(H, I)** Quantitative comparisons of α-tubulin in cells treated with nocodazole **(H)** or monastrol **(I)** for 1 h. Data represent the mean ± SD relative intensity. *** indicates p < 0.005 (n = ~30).

Next, we examined whether centrosome-free MT asters were maintained after mitotic spindle dissolution and whether MT elongation activity was retained in an MT regrowth assay. In control cells, MTs were completely disrupted by cold treatment for 30 min (Fig 6A-a), and MT initiation from centrosomes was observed within 3 min after transfer to pre-warmed media (Fig 6B-a). After 25 min, the majority of cells showed bipolar spindles (Fig 6C-a). In TrAP-CSAP-overexpressing cells, MTs and centrosome-free MT asters were detected immediately after cold treatment (Fig 6A-b; quantitative data, Fig 6A-c), suggesting that excess CSAP inhibits MT depolymerization. Next, we performed immunofluorescence microscopy to observe cold-treated cells stained with the SBP antibody targeting the TrAP tag. TrAP-CSAP was observed on centrosome-free MT asters and cold-resistant MTs (Fig 6D), suggesting that MT depolymerization was inhibited by CSAP binding. In addition, MT initiation from centrosome-free MT asters was observed within 3 min after transfer to pre-warmed media (Fig 6B-b and 6B-c). After 25 min, MTs elongating from centrosome-free MT asters exhibited multipolar spindle formation (Fig 6C-b), indicating that they possessed the same capacity as centrosomes for MT elongation and mitotic spindle formation. α-Tubulin analysis suggested more extensive MT formation in CSAP-overexpressing cells (Fig 6C-c). To establish inhibition of MT depolymerization by CSAP binding, we performed anti-α-tubulin immunostaining in GFP-CSAP-overexpressing cells after 1 h of nocodazole treatment (Fig 6E). In this experiment, cells were fixed with 4% PFA in the presence of nocodazole. We observed centrosome-free MT asters and short MTs elongating from them; GFP-CSAP co-localized with centrosome-free MT asters and MTs as confirmed by quantitative analysis of α-tubulin staining after 1 h of nocodazole treatment (Fig 6E and 6H). However, centrosome-free MT asters were not detected in mitotic cells treated with nocodazole for 12 h until cell fixation (Fig 6F). Several centrosome-free MT asters that formed in the presence of monastrol exhibited stronger α-tubulin association; they were close to the central domain of the cell, i.e., around the centrosomes, and the cells appeared to have a mono-polar spindle (Fig 6G and 6I).

## Discussion

In this study, we showed that spindle association with excess CSAP caused formation of centrosome-free MT asters, in which γ-tubulin and CDK5RAP2 were not detected, a phenotype not reported in a previous study on CSAP function [1]. Recruitment of PCM proteins such as Aurora A and pericentrin may contribute to the centrosomal activity of centrosome-free MT asters, allowing them to grow MTs that can bind kinetochores and align chromosomes in mitosis. Because centrosome-free MT aster formation in mitosis causes catastrophic cell division (Fig 2B), we presume that cells strongly expressing GFP-CSAP would be eliminated by selection. Our data show that abnormal expression of CSAP causes defects in MT dynamics during mitosis. The MT-instability and NuMA mislocalization in CSAP-depleted cells produce a multipolar spindle phenotype (Fig 5E). We suggest that CSAP plays a role in MT stabilization and that this stabilization involves the maintenance of NuMA at the spindle poles. This conclusion is supported by the enhanced MT formation in mitotic spindles and the significant accumulation of NuMA in CSAP-overexpressing cells (Fig 4A and 4B). However, this phenotype appears different in terms of the formation of centrosome-free MT asters. We thus concluded that these two multipolarities might be mechanically different. These findings suggest that binding of CSAP to centrosomes and the spindle around centrosomes during mitosis inhibits MT depolymerization, thereby stabilizing the mitotic spindle. Because CSAP is localized at centrosomes and mitotic spindles around the poles [1], we suspect that excess CSAP levels lead to highly stabilized MTs, resulting in centrosome-free MT aster formation on the mitotic spindle. Unusual NuMA staining has been observed in cells microinjected with the monoclonal polyglutamylation modification antibody GT335 [14]. It is thus possible that enhanced polyglutamylation induced by CSAP association recruited NuMA to the centrosomes and centrosome-free MT asters. NuMA associates with dynein, which transports it towards MT minus-ends and deposits it at spindle poles [32,33]. Dynein associates with acetylated tubulin [49]. However, polyglutamylation directly regulates the MT-dynein interaction in *Chlamydomonas reinhardtii* [50].

CSAP associates with polyglutamylated MTs around centrosomes during mitosis [1]; our data suggest that CSAP stabilizes mitotic spindles by inhibiting MT depolymerization. We speculate that CSAP localization at centrosomes and aster MTs is essential for proper bi-polar spindle formation. MT minus-end binding factors such as EB1 and TACC3 play critical roles in MT stabilization [51–54]; however, MT stabilization by CSAP is a unique pathway that occurs through tubulin modification, which is not well understood. Polyglutamylation on the MT appears to produce a positive feedback effect on CSAP association. However, polyglutamylation of aster MTs did not change after CSAP overexpression or CSAP depletion. Remarkably, significant polyglutamylation was observed in CSAP-depleted cells, indicating that CSAP is not required for initial polyglutamylation at the centrosome. We conclude that the excess polyglutamylation after CSAP overexpression is a downstream effect of MT stability. We speculate that CSAP-mediated microtubule regulation in cells involved in brain development (where high levels of tubulin polyglutamylation are observed [5]) is different from that in cultured cells. CSAP localization on centrioles in mitotic centrosomes has been reported [1]; we therefore suggest that CSAP has another function in centrioles.

## Supporting Information

S1 Movie Mitosis progression of control U2OS cells expressing GFP–α-tubulin and H2B-mRFP. (MOV)

S2 Movie Mitosis progression of CSAP-overexpressing U2OS cells expressing GFP–α-tubulin and H2B-mRFP. (MOV)

S3 Movie Mitosis progression of CSAP-overexpressing U2OS cells expressing GFP–α-tubulin and H2B-mRFP. (MOV)

S4 Movie Mitosis progression of CSAP-overexpressing U2OS cells expressing GFP–α-tubulin and H2B-mRFP. (MOV)
